# An exploratory cluster-randomized controlled trial on mindfulness yoga’s effectiveness in school-refusing children: reductions in SCAS-C physical injury fears and pulse rate

**DOI:** 10.3389/fnhum.2024.1468729

**Published:** 2024-12-11

**Authors:** Suguru Kawazu, Marie Amitani, Hajime Suzuki, Haruka Amitani, Takako Monuki, Midori Wada, Satomi Toyohira, Kazumasa Hamada, Takako Yamamoto, Takuya Yoshimura, Kimiko Mizuma, Yuko Nishida, Hiroko Watanabe, Masayuki Hirose, Koshiro Tagawa, Keiko Ota, Akihiro Asakawa, Tetsuhiro Owaki

**Affiliations:** ^1^Department of Psychosomatic Internal Medicine, Kagoshima University Graduate School of Medical and Dental Sciences, Kagoshima, Japan; ^2^Education Center for Doctors in Remote Islands and Rural Areas, Kagoshima University Graduate School of Medical and Dental Sciences, Kagoshima, Japan; ^3^Department of Community-Based Medicine, Kagoshima University Graduate School of Medical and Dental Sciences, Kagoshima, Japan; ^4^Department of Oral and Maxillofacial Surgery, Kagoshima University Graduate School of Medical and Dental Sciences, Kagoshima, Japan; ^5^Multidisciplinary Pain Center, Kyushu University Hospital, Fukuoka, Japan; ^6^Center for Clinical and Translational Research, Kyushu University Hospital, Fukuoka, Japan; ^7^Center for Clinical Research and Innovation, Osaka City University Hospital, Osaka, Japan

**Keywords:** school refusal, mindfulness yoga, anxiety reduction, cognitive behavioral therapy, pediatric mental health

## Abstract

**Introduction:**

School refusal is one of the serious problems with children’s mental health, and various studies have examined its prevalence and factors among students. Although many studies suggested that anxiety and depression are deeply associated with school refusal, there is little agreement as to effective interventions. The purpose of this paper is to evaluate the efficacy and safety of mindfulness yoga intervention in children with school refusal.

**Method:**

This study is a multicenter, exploratory, open cluster-randomized controlled trial. 43 participants aged 10–15 years with school refusal were randomly assigned to a non-yoga group with treatment as usual (TAU) which includes cognitive behavioral therapy based on self-monitoring, or a yoga group (4-week mindfulness yoga program provided by video sessions + TAU). The primary outcome was symptoms of anxiety evaluated by Spence Children’s Anxiety Scale-Children (SCAS-C). Participants were assessed in four time periods: a 2-week baseline (Day −14), a baseline (Day 1), a post-test after 4 weeks of treatment (Day 29), and an 8-week follow-up (Day 85). Statistical analysis was conducted by a linear mixed effect model using SAS version 9.4.

**Results:**

43 participants were included in the Full-analysis set (FAS) (21 in the mindfulness yoga group and 22 in the non-yoga group). The estimates of SCAS-C at post-test adjusted for baseline values in each treatment group were 39.9 in the mindfulness yoga group and 39.4 in the non-yoga group. The between-group difference for the estimates was 0.4 (80%CI −4.8 to −5.6, *p* = 0.54), which indicated mindfulness yoga program has no significant effect on anxiety compared with TAU. However, on an exploratory analysis of the subscale of SCAS-C, significant improvement was observed on the Physical Injury Fears subscale. The pulse rate was significantly lower in the yoga group compared to the non-yoga group.

**Conclusion:**

This study indicated the safety of a mindfulness yoga intervention for children with school refusal, but the effectiveness of the intervention for anxiety was limited. Further research is needed to determine the long-term effects of yoga and how it can best be integrated with other therapies.

## 1 Introduction

Regular school attendance is critical to the growth and development of children and adolescents (McConnell and Kubina, 2014), but school refusal, in which children repeatedly avoid school due to psychological distress, causes significant problems (Maynard et al., 2018). This problem not only hinders academic progress but also increases the likelihood of early dropout. In Japan, the Ministry of Education, Culture, Sports, Science and Technology (MEXT) defines school refusal as students who are absent from school for 30 or more days per year due to psychological, emotional, physical, or social factors, excluding absences due to illness or economic reasons (MEXT: Ministry of Education, Culture, Sports, Science and Technology, Japan, 1998). School refusal in Japan is an important concern, and various studies have examined its prevalence and factors among students. MEXT survey (MEXT: Ministry of Education, Culture, Sports, Science and Technology, Japan, 2023) found that 3.2% of children enrolled in elementary and junior high schools in Japan are truant.

Given the complexity of school refusal behavior involving many interrelated factors. However, the treatment situation of children who refuse to attend school is complex, dealing with a variety of psychological and cognitive challenges, and requires tailored interventions (Heyne et al., 2021). Traditional treatments such as cognitive behavioral therapy (CBT) are commonly used but do not fully address the needs of all children who refuse to attend school not be addressed (Dummett, 2010), CBT typically includes elements such as psychoeducation, relaxation techniques, cognitive restructuring, graded exposure, and social skills training (Kearney and Albano, 2007). However, randomized controlled trials (RCTs) evaluating the effectiveness of CBT have been limited and only partially successful. This has led to the study of alternative therapies such as mindfulness yoga.

Recent studies highlight the potential of mindfulness and yoga to improve children’s psychological resilience and emotional wellbeing. Mindfulness yoga combines traditional yoga postures with mindfulness techniques, offering a comprehensive approach to alleviating psychological issues (La Torre et al., 2020). By integrating physical movements with a focus on present-moment awareness, this practice promotes emotional stability and stress management (La Torre et al., 2020). It is particularly suitable for school environments, incorporating it into daily routines and providing ongoing support for children (Razza et al., 2013). Although mindfulness interventions generally reduce anxiety and stress in children, the specific context of school refusal requires targeted research on these therapies (Noggle et al., 2012). Mindfulness-based approaches have been shown to enhance attention, executive functioning, and self-regulation, which are crucial for managing school-related stress (Razza et al., 2013; Mak et al., 2018). Additionally, mindfulness programs have proven effective in improving self-regulation and focus on preschool children, which is critical for dealing with school refusal (Razza et al., 2020). Incorporating yoga and mindfulness into educational settings has shown improvements in emotional and psychosocial wellbeing, directly addressing anxiety symptoms (Bazzano et al., 2018). Moreover, incorporating mindfulness-based cognitive therapy, which has demonstrated effectiveness in reducing anxiety among children, could yield more conclusive insights into the role of mindfulness in managing school refusal (Shetty et al., 2020).

Although many studies suggested that anxiety and depression are deeply associated with school refusal, there is little agreement as to effective interventions. The purpose of this study was to develop a mindfulness yoga program that children can do alone at home and to test its effectiveness on anxiety in children who are not attending school (Amitani et al., 2022).

## 2 Materials and methods

### 2.1 Study design

This study employs a multi-center, exploratory, cluster randomized controlled design to reduce the risk of bias due to environmental and social influences on participants. A block randomization method was applied, with two strata: “free schools” and “facilities other than free school.” The allocation ratio was 1:1. In this design, facilities, including schools, were randomly assigned as clusters to either a non-yoga group with treatment as usual (TAU) which includes CBT based on self-monitoring, or a yoga group (4-week mindfulness yoga program provided by video sessions + TAU).

### 2.2 Participants and exclusion criteria

**Figure 1:**
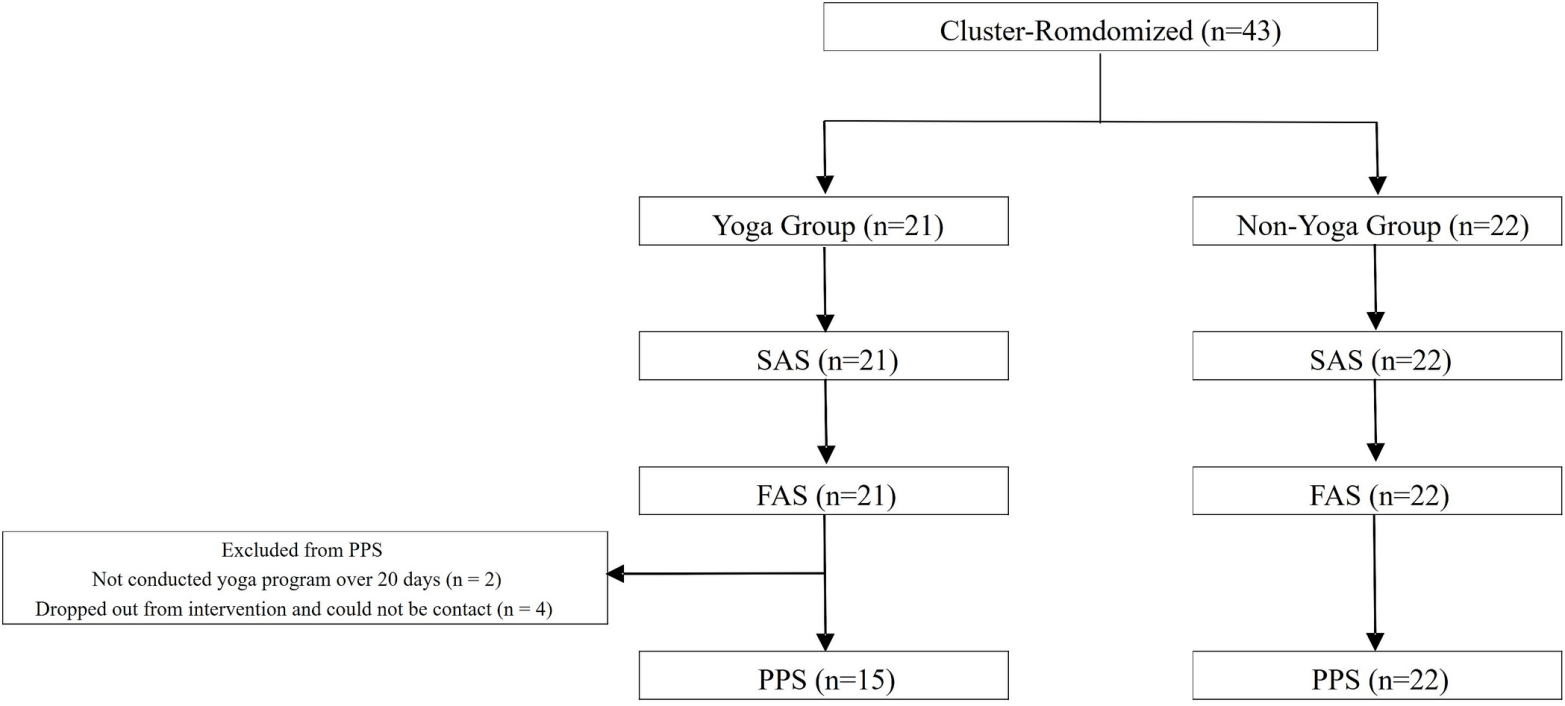
Participant flow and population in each analysis set. The population for analysis was divided into a Safety analysis set (SAS), Full-analysis set (FAS) and Per-protocol set (PPS) according to the criteria defined in the protocol article. In this study, SAS was used to evaluate safety, FAS was used to analyze the primary and secondary endpoints, and PPS was used to confirm the robustness of the analysis results for the primary endpoint.

Children aged 10–15 years with no continuous yoga experience who met the criteria for school refusal (absence from school for at least 30 days) were recruited from the public. Exclusion criteria were detailed as follows: children with an overall intelligence quotient (IQ) of 70 or less on the Wechsler Intelligence Scale for Children, Fourth Edition (WISC-IV). Children who have been exposed to abuse or bullying with the potential for self-harm or other harm, and who are identified as requiring immediate social attention. Children with a history of schizophrenia, paranoid disorder, or depression with thoughts of death. Consent to participate in the study was obtained from 43 participants who did not meet the exclusion criteria and were found to be eligible for the study (Figure 1). The sample size calculation prior to study initiation assumed a between-group difference of 13.7 in SCAS-C scores between the yoga and non-yoga groups, with 43 participants providing 83% statistical power. All participants were then assigned by cluster randomization, 21 to the yoga group and 22 to the non-yoga group. The population for analysis was divided into a Safety analysis set (SAS), Full-analysis set (FAS) and Per-protocol set (PPS) according to the criteria defined in the protocol article (Amitani et al., 2022). In this study, SAS was used to evaluate safety, FAS was used to analyze the primary and secondary endpoints, and PPS was used to confirm the robustness of the analysis results for the primary endpoint. In the yoga group, the number of participants in each analysis group was 21 for SAS, 21 for FAS, and 15 for PPS (6 excluded from PPS); in the non-yoga group, 22 for SAS, 22 for FAS, and 22 for PPS. The 6 PPS exclusions in the yoga group included 2 because the mindfulness yoga program had not been implemented for more than 20 days, and the remaining 4 because the participants did not wish to continue participating (Avoidance of medical visits due to COVID-19, family convenience). In the FAS, the distribution of school types was as follows: in the yoga group, 4 participants (19.0%) were from elementary schools and 17 (81.0%) from middle or high schools; in the non-yoga group, 5 participants (22.7%) were from elementary schools and 17 (77.3%) from middle or high schools. Regarding facility types, the yoga group comprised 0 participants (0.0%) from free schools and 21 (100.0%) from non-free school facilities, while the non-yoga group included 3 participants (13.6%) from free schools and 19 (86.4%) from non-free school facilities.

### 2.3 Intervention

#### 2.3.1 Development of the mindfulness yoga program

The mindfulness yoga program was specially designed with input from children of the same age group to encourage regular implementation. The program is a four-week program that gradually increases the intensity of the intervention from week 1 to week 4. The first week of the program includes basic breathing exercises, basic yoga poses, and brief body scan meditation. As the program progressed, we incorporated more complex yoga poses. Duration of intervention time was approximately 15–20 min per day throughout the intervention period. (Supplementary material 1b–e). The yoga intervention in this study was delivered with emphasis on participant autonomy, based on the principle that maximizing the benefits of yoga practice without engaging in evaluative or comparison such as requirement of “practicing every day” or “doing all the programs. Therefore, the instruction on the program was emphasized focusing on savoring “one’s own physical sensations” not to evaluate the “appropriateness” of the poses, which leads to feelings of self-acceptance (i.e., accepting himself/herself “as they are”).”

#### 2.3.2 Details of interventions in the treatment groups

**Figure 2:**
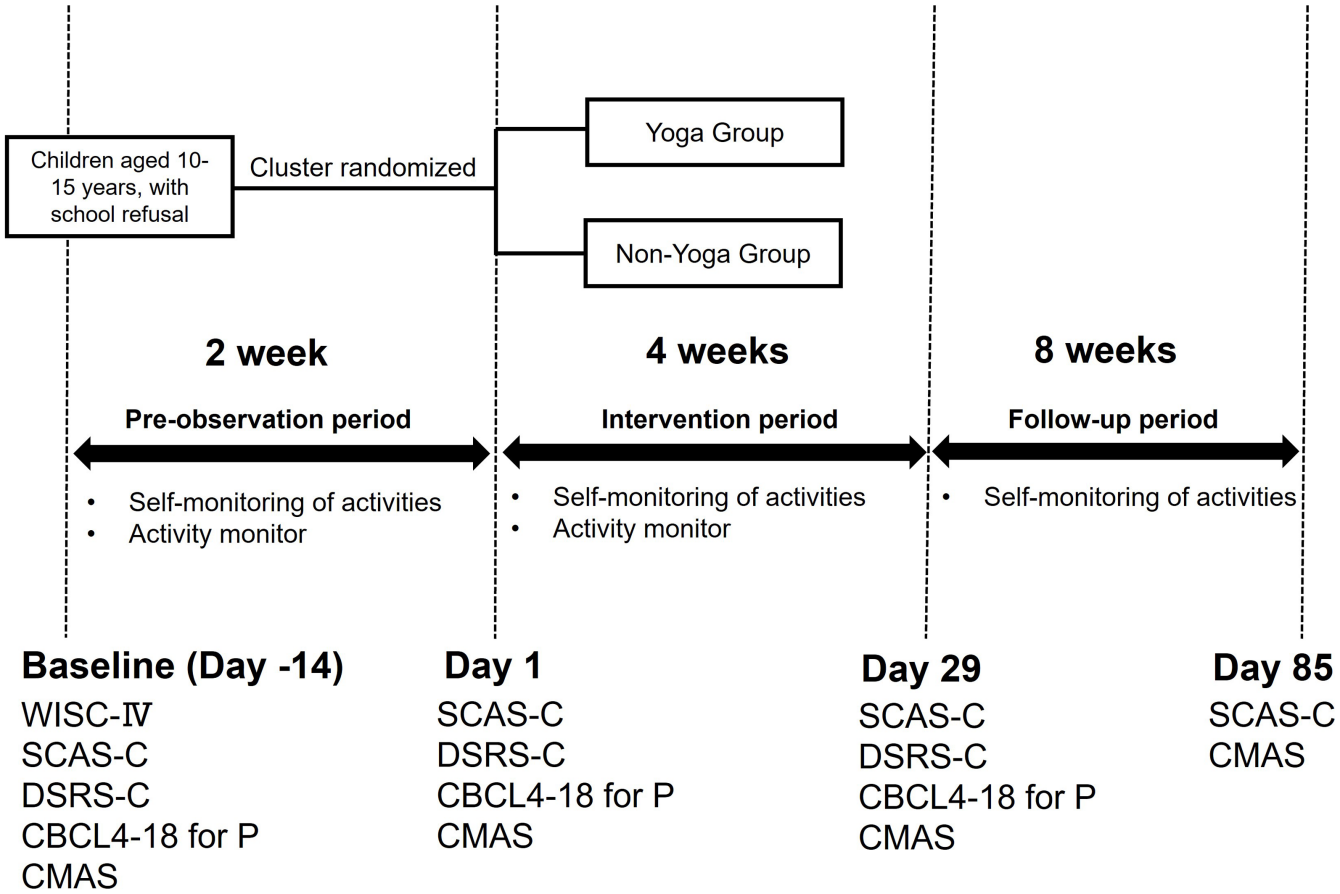
Research diagram. This figure shows the study flow and interventions. Yoga Group: Yoga + TAU, Non-Yoga Group: TAU. TAU (Treatment as Usual): 30-min CBT session consisting of self-monitoring and lifestyle guidance.

Figure 2 shows the flow of the study.

Yoga group: Participants in this group underwent the four-week mindfulness yoga program in addition to TAU, which consisted of a video. The participants performed yoga every day for 4 weeks with no set amount of time per day; those who did not perform the yoga program for more than 20 days were excluded from the PPS. In order to monitor their mindfulness yoga practice, they are asked to keep a mindfulness yoga diary (Supplementary material 2a–d) after they did the mindfulness yoga practice. The participants and their parents were provided a face-to-face instruction about the mindfulness yoga by researchers using the yoga video for Day 1 (Supplementary material 1a). To support continued engagement, regular interviews and telephone consultations were conducted throughout the intervention period to provide assistance with tips on implementation and address concerns. However, considering the potential psychological burden on participants, no additional monitoring of practice adherence (such as requesting parental verification of practice) was implemented in this study. The further details of the program are as described in the previously presented protocol paper (Amitani et al., 2022).

Non-yoga group: Participants in this group received a 30-min CBT session consisting of self-monitoring and lifestyle guidance as TAU.

#### 2.3.3 Self-monitoring of activities

All of the participants kept a daily record of their day-to-day activities (the time they woke up, went to bed, and went to school) for 14 weeks (Day −14 ~ Day 85), which were used to collect information regarding school attendance. Lifestyle guidance and CBT were provided using their self-monitoring data.

### 2.4 Primary and secondary outcomes

#### 2.4.1 Psychological testing

Anxiety was assessed using the Spence Children’s Anxiety Scale-Children (SCAS-C) as the primary outcome. Secondary psychological assessments included the Depression Self-Rating Scale for Children (DSRS-C), the Child Behavior Checklist for Ages 4–18 Parent Report (CBCL4-18 for P), and the Children’s Manifest Anxiety Scale (CMAS). The SCAS-C and CMAS were completed by the participants themselves on Day -14, Day 1, Day 29 and Day 85 and DSRS-C was completed by the participants themselves on Day -14, Day 1 and Day 29. The CBCL4-18 for P was answered by the participants’ parents on Day −14, Day 1, and Day 29.

#### 2.4.2 Basic data

Blood pressure, pulse, temperature, height, and weight were measured on Day −14, Day 1, Day 29 and Day 85.

#### 2.4.3 Activity and sleep cycle

All of the participants wore an accelerometry-based activity monitor MicroTag MTN-221 (ACOS Co, Ltd., Nagano, Japan) on their waist 24 h a day for 6 weeks (Day −14 ~ Day 29). The actigraph data were assessed using the algorithm supplied by the SleepSign©Act Ver2.0 software (Kissei Comtec, Matsumoto, Japan). We evaluated daily activity, sleep cycle, and sleep efficiency (total sleep time/time in bed × 100).

#### 2.4.4 Ratio of school attendance

School attendance days were extracted from the self-monitoring and the percentage of school attendance days was calculated (ratio of school attendance = days of school attended/the days participant should have attended school × 100).

### 2.5 Safety assessment

Safety was assessed by recording the incidence of adverse events, serious adverse events, and side effects in the yoga and non-yoga groups.

### 2.6 Statistical analysis

Data were analyzed using mixed-effects models to accommodate the clustered design of the trial. Analyses controlled for treatment groups, baseline values and stratification factors (type of facility: free schools/non-free school facilities). The cluster-randomized structure was factored into the analysis to ensure reliable and applicable results across different settings. Analyses followed the intent-to-treat principle, and missing values were not supplemented due to the exploratory nature of this study. No adjustment for multiplicity was made. Baseline data were the most recent data before the intervention.

Differences between groups were evaluated using independent t-tests for continuous variables and chi-squared tests for categorical variables. A repeated measures ANOVA assessed changes over time within and between groups. We used a mixed-effects model with Restricted Maximum Likelihood estimation to analyze both fixed and random effects. Fixed effects were evaluated using Type III sum of squares, and random effects assessed individual variability through standard deviation and variance calculations. Geisser–Greenhouse corrections adjusted for sphericity violations in F-tests.

A chi-square test confirmed the effectiveness of our participant matching procedure. Longitudinal changes were analyzed using the Holm-Šídák method to adjust for multiple comparisons, ensuring the statistical integrity of our findings throughout the study.

Statistical analyses were conducted using SAS Version 9.4 for Windows. Since this was an exploratory study, the significance level of the test was 10% two-sided for the primary endpoint, 20% two-sided for the secondary endpoints, and the confidence coefficient for interval estimation was 80% two-sided.

## 3 Results

### 3.1 Background and basic date of the participants

The background and basic date of the participants are shown in Table 1.

**Table 1:** Study participants at baseline.

		Yoga group			Non-yoga group			Overall	
	*n*	Mean	SD	*n*	Mean	SD	*n*	Mean	SD
Age	21	13.0	1.3	22	13.2	1.6	43	13.1	1.5
Height (cm)	21	156.6	9.0	22	154.1	7.0	43	155.3	8.0
Weight (kg)	21	46.3	9.5	22	49.3	11.4	43	47.8	10.5
Body mass index (kg/m^2^)	21	18.7	2.7	22	20.7	4.1	43	19.7	3.6
Systolic blood pressure (mmHg)	21	102.0	10.7	22	103.3	14.9	43	102.7	12.9
Diastolic blood pressure (mmHg)	21	60.5	9.6	22	62.1	12.3	43	61.3	11.0
Pulse rate (bpm)	21	75.5	8.7	22	79.1	12.6	43	77.3	10.9
Body temperature (°C)	21	36.4	0.3	19	36.4	0.3	40	36.4	0.3
SCAS-C	21	38.6	21.2	22	47.9	22.7	43	43.3	22.2
DSRS-C	21	17.4	6.7	22	18.5	7.2	43	18.0	6.9
CBCL4-18 for P, T score	21	62.5	8.5	22	65.8	9.0	43	64.2	8.8
CMAS, A scale total	21	22.3	8.8	21	23.4	9.5	42	22.8	9.1
Daily activity (kcal)	15	1,698.7	223.3	18	1,804.4	264.8	33	1,756.3	248.8
Sleep cycle (h)	13	24.2	0.4	19	24.0	0.3	32	24.1	0.4
Sleep efficiency (%)	15	77.7	7.2	18	77.1	8.4	33	77.4	7.7

	Yoga group, *n* (%)	Non-yoga group, *n* (%)	Overall, *n* (%)
Sex			
Female	13 (61.9)	14 (63.6)	27 (62.8)
Male	8 (38.1)	8 (36.4)	16 (37.2)
Types of schools attended			
Elementary school	4 (19.0)	5 (22.7)	9 (20.9)
Junior high school	17 (81.0)	17 (77.3)	34 (79.1)
Types of facility			
Free school	0 (0.0)	3 (13.6)	3 (7.0)
Non-free school	21 (100.0)	19 (86.4)	40 (93.0)
Opportunity to participate			
Family	13 (61.9)	11 (50.0)	24 (55.8)
School	5 (23.8)	2 (9.1)	7 (16.3)
Homeroom teacher	3 (14.3)	6 (27.3)	9 (20.9)
Free school	0 (0.0)	1 (4.5)	1 (2.3)
Poster	3 (14.3)	6 (27.3)	9 (20.9)

### 3.2 Primary outcome

#### 3.2.1 SCAS-C scores in all participants

**Figure 3:**
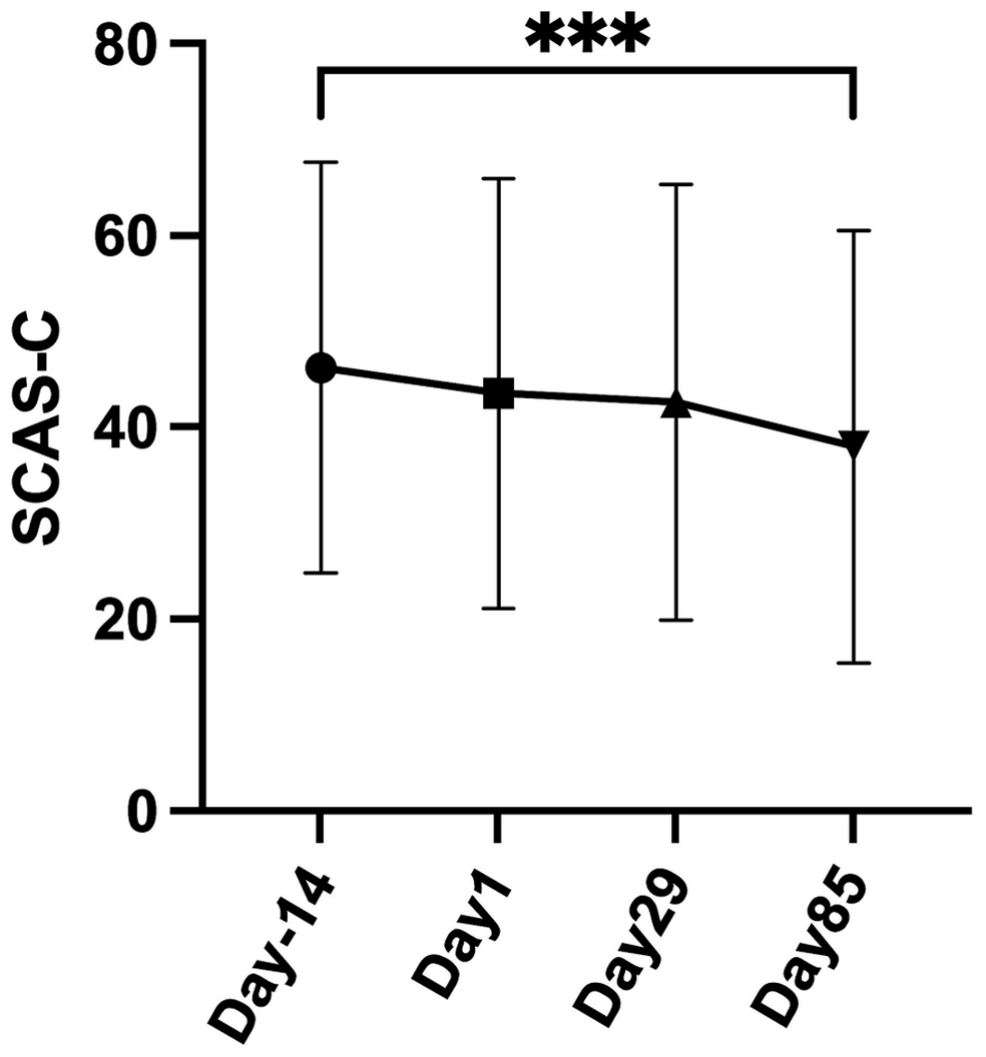
Longitudinal effects of treatment on SCAS-C scores using mixed-effects model in all participants. This figure illustrates the SCAS-C scores in all participants, as determined by longitudinal mixed-effects modeling in the Full-analysis set (FAS) (*n* = 43) (mean difference = 8.256, adjusted *p* = 0.0006).

SCAS-C scores for all participants in the yoga and non-yoga groups combined (*n* = 43 in the FAS) demonstrated a statistically significant difference between Day −14 and Day 85 (Figure 3). Longitudinal analysis utilizing mixed-effects modeling, with F-statistics for the numerator and denominator degrees of freedom of 2.619 and 101.3, respectively, and a *p*-value of 0.0016, corroborated a significant treatment effect on SCAS-C scores in the study population. Inter-individual variability was substantial, with a standard deviation of 20.05 and a variance of 402.0. Residual effects exhibited considerable variation, as indicated by a standard deviation of 8.799 and a variance of 77.42. Adjustments for sphericity violation were necessitated, as reflected by Geisser–Greenhouse’s epsilon of 0.8729. The efficacy of the matching process was validated by a chi-square test, yielding a value of 167.4 with one degree of freedom and a *p*-value <0.0001. Longitudinal comparisons revealed significant differences, particularly between Day −14 and Day 85, with a mean difference of 8.256 and an adjusted *p*-value of 0.0006.

#### 3.2.2 Comparison of adjusted SCAS-C scores between yoga and non-yoga groups

**Figure 4:**
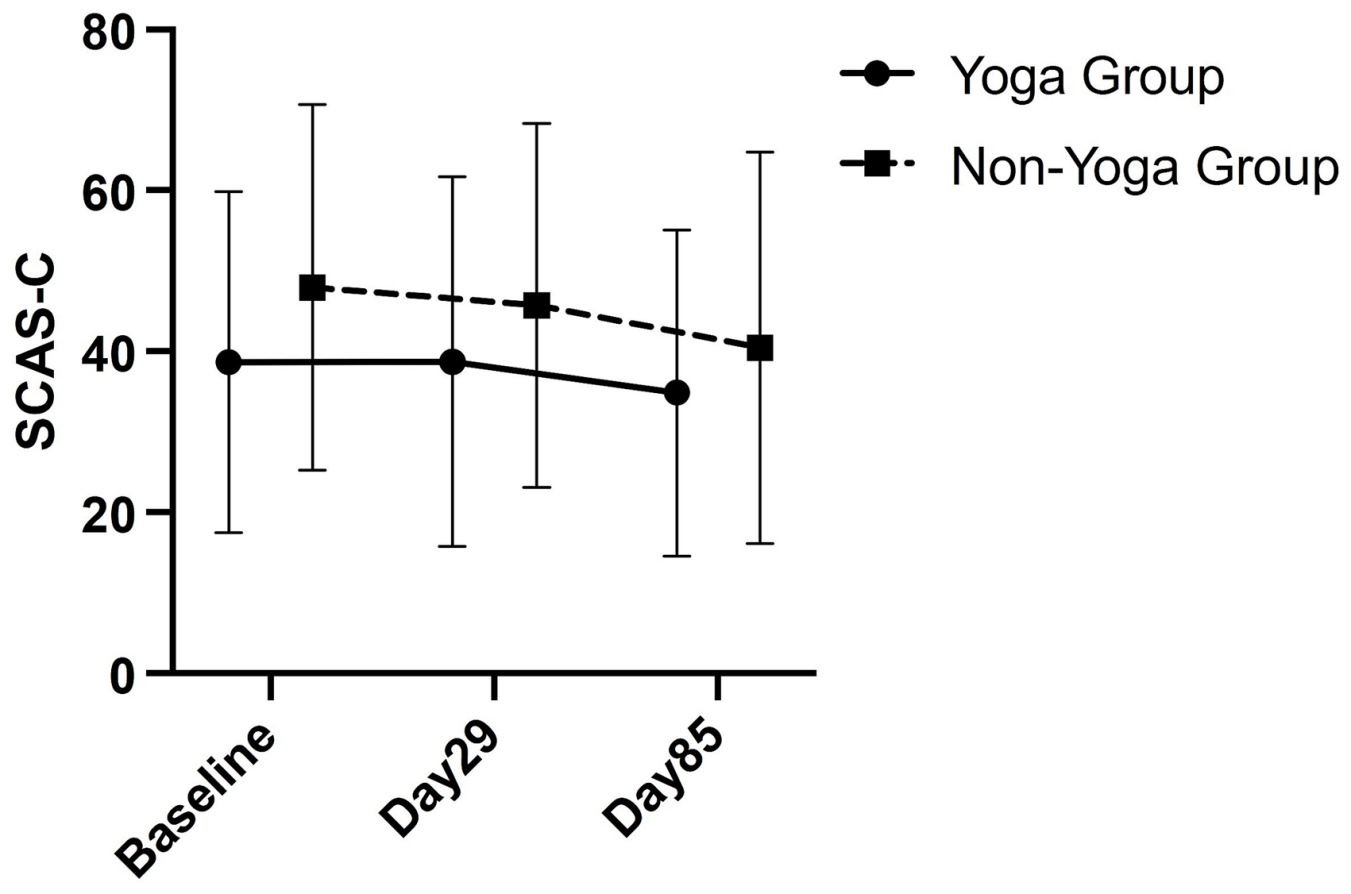
Comparison of yoga and non-yoga groups on SCAS-C. This figure shows a comparison of SCAS-C between the yoga and non-yoga groups on Day 29 and Day 85 in a mixed-effects modeling analysis in the Full-analysis set (yoga group *n* = 21, non-yoga group *n* = 22).

Figure 4 illustrates a comparison of SCAS-C scores, with treatment groups, baseline values and facility as covariates, between the yoga and non-yoga groups on Day 29 and Day 85 using mixed-effects modeling analysis in the FAS (yoga group *n* = 21, non-yoga group *n* = 22). The results indicate adjusted SCAS-C scores for the yoga group (38.6) and the non-yoga group (39.0) on Day 29. The inter-group difference was −0.5 with an 80% confidence interval of [−5.9, 5.0], which was not statistically significant (one-sided *p* = 0.4574). On Day 85, the yoga group scored 34.7 and the non-yoga group 33.7, with an inter-group difference of 1.0. The 80% confidence interval for this difference was [−4.6, 6.6], and it was also not statistically significant (one-sided *p* = 0.5924). The intraclass correlation coefficient was 0.28.

#### 3.2.3 SCAS-C subscales

Table 2 shows a comparison of the SCAS-C subscale between the yoga and non-yoga groups on Day 29 in a mixed-effects modeling analysis in the FAS (yoga group *n* = 21, non-yoga group *n* = 22).

**Table 2:** Comparison of yoga and non-yoga groups on SCAS-C subscale.

	Yoga group (Mean)	Non-yoga group (Mean)	Mean difference	Std. Error	80% CI	*p*-value
Separation anxiety	4.9	4.1	0.8	0.8	−0.2, 1.9	0.30
Social phobia	8.3	8.0	0.3	1.1	−1.2, 1.7	0.82
Obsessive-compulsive disorder	6.3	6.4	−0.1	0.9	−1.3, 1.1	0.90
Panic-agoraphobia	7.7	6.8	0.9	1.3	−0.8, 2.6	0.51
Generalized anxiety	7.0	7.8	−0.8	1.4	−2.6, 1.0	0.55
Fears of physical injury	5.3	6.3	−1.0	0.6	−1.7, −0.2	0.11^*^

This table shows a comparison of the SCAS-C subscale between the yoga and non-yoga groups on Day 29 in a mixed-effects modeling analysis in the Full-analysis set (FAS) (yoga group *n* = 21, non-yoga group *n* = 22).*Fear of physical injury subscale showed a significant reduction in the yoga group versus the control group. The estimated mean difference was −1.0 (SE = 0.6), with 80% confidence intervals ranging from −1.7 to −0.2, indicating statistical significance (*p* = 0.11). Statistical analyses were conducted using SAS Version 9.4 for Windows. Since this was an exploratory study, the significance level of the test was 10% two-sided for the primary endpoint, 20% two-sided for the secondary endpoints, and the confidence coefficient for interval estimation was 80% two-sided.

In a mixed-effects model analysis comparing the effects of the yoga intervention on the SCAS-C subscales with treatment groups, baseline values and facility as covariates, only the fear of physical injury subscale showed a significant reduction in the yoga group versus the control group. The estimated mean difference was −1.0 (SE = 0.6), with 80% confidence intervals ranging from −1.7 to −0.2, indicating statistical significance (*p* = 0.11). No significant differences were found for the other subscales, including separation anxiety, social fear, obsessive-compulsive disorder, panic/square fear, and generalized anxiety.

### 3.3 Secondary outcomes

#### 3.3.1 Psychosomatic tests

Table 3 shows a comparison of DSRS-C, CBCL 4–18 for P, CMAS between the yoga and non-yoga groups on Day 29 in a mixed-effects modeling analysis in the FAS (yoga group *n* = 21, non-yoga group *n* = 22).

**Table 3:** Comparison of yoga and non-yoga groups on DSRS-C, CBCL 4–18 for P and CMAS.

	Yoga group (Mean)	Non-yoga group (Mean)	Mean difference	Std. Error	80% CI	*p*-value
DSRS-C	18.9	17.7	1.1	1.5	−0.9, 3.1	0.46
CBCL4-18 for P	64.1	64.4	−0.3	2.8	−4.0, 3.4	0.92
CMAS	21.9	22.0	−0.1	1.8	−2.5, 2.3	0.96

This table shows a comparison of DSRS-C, CBCL 4–18 for P, CMAS between the yoga and non-yoga groups on Day 29 in a mixed-effects modeling analysis in the Full-analysis set (FAS) (yoga group *n* = 21, non-yoga group *n* = 22).

In DSRS-C, CBCL4-18 for P, and CMAS, the *p*-values were 0.46, 0.92, and 0.96, respectively, and mixed effects models revealed no significant differences between the intervention and control groups (Table 3).

#### 3.3.2 The ratio of school attendance

Table 4 shows the estimated percentage of days in school for yoga and non-yoga groups over two periods. For Days 2–29, the yoga group’s mean attendance was 31.36% compared to 37.87% for the non-yoga group, with a mean difference of −6.51% (SE: 9.60, 80% CI: [−19.05, −6.02], *p* = 0.50) in the FAS (yoga group *n* = 21, non-yoga group *n* = 22). For Days 30–85, the yoga group’s mean attendance was 41.09% versus 42.57% for the non-yoga group, with a mean difference of −1.48% (SE: 10.01, 80% CI: [−14.53, 11.58], *p* = 0.88) in the FAS (yoga group *n* = 21, non-yoga group *n* = 22). There were no significant differences between the yoga and non-yoga groups.

**Table 4:** Comparison of estimated school attendance rates between yoga and non-yoga groups from day 2 to day 85.

	Yoga group (Mean)	Non-yoga group (Mean)	Mean difference	Std. Error	80% CI	*p*-value
Day 2–Day 29	31.36	37.87	−6.51	9.60	−19.05, −6.02	0.50
Day 30–Day 85	41.09	42.57	−1.48	10.01	−14.53, −11.58	0.88

This table presents the estimated percentage of days in school for yoga and non-yoga groups over two periods in the Full-analysis set (FAS) (yoga group *n* = 21, non-yoga group *n* = 22).

#### 3.3.3 Others

Table 5 shows a comparison of activity, sleep cycle, and vital signs between the yoga and non-yoga groups on Day 29 in a mixed-effects modeling analysis in the FAS (yoga group *n* = 21, non-yoga group *n* = 22).

**Table 5:** Comparison of yoga and non-yoga groups on activity, sleep cycle, and vital sign.

	Yoga group (Mean)	Non-yoga group (Mean)	Mean difference	Std. Error	80% CI	*p*-value
Daily activity (kcal)	1,914.9	1,876.1	38.7	70.5	−54.9, 132.2	0.59
Sleep cycle (s)^1^	5.7^2^	6.3^2^	−0.5^3^	1.5^3^	−2.5, 1.4^3^	0.72
Sleep efficiency (%)	77.7	75.8	1.9	2.3	−1.3, 5.0	0.43
Body temperature (°C)	36.2	36.3	−0.1	0.1	−0.3, 0.0	0.32
Systolic blood pressure (mmHg)	101.7	102.8	−1.2	3.1	−5.4, 3.0	0.71
Diastolic blood pressure (mmHg)	64.1	66.9	−2.8	3.7	−7.6, 2.0	0.45
Pulse rate (bpm)	75.6	81.3	−5.7	3.0	−9.6, −1.8	0.07^*^
Weight (kg)	50.2	49.0	1.1	1.1	−0.3, 2.6	0.32
Body mass index (kg/m^2^)	20.6	20.2	0.4	0.4	−0.1, 1.0	0.32

This table shows a comparison of activity, sleep cycle, and vital signs between the yoga and non-yoga groups on Day 29 in a mixed-effects modeling analysis in the Full-analysis set (FAS) (yoga group *n* = 21, non-yoga group *n* = 22).

1 Mixed-effects model analysis of the logarithmically transformed absolute values of the difference between sleep cycle duration and 24 h, from the 22nd to the 29th day.

2 Time displayed as logarithmic values (unit: seconds).

3 Ratio of time for yoga group compared to non-yoga group, expressed as logarithmic values (unit: seconds).

*The mean pulse rate in the yoga group was 75.6 bpm compared to 81.3 bpm in the non-yoga group, with a mean difference of −5.7 bpm (standard error = 3.0, 80% CI: [−9.6, −1.8], *p* value = 0.07).

Statistical analyses were conducted using SAS Version 9.4 for Windows. Since this was an exploratory study, the significance level of the test was 10% two-sided for the primary endpoint, 20% two-sided for the secondary endpoints, and the confidence coefficient for interval estimation was 80% two-sided.

The pulse rate was significantly lower in the yoga group compared to the non-yoga group. The mean pulse rate in the yoga group was 75.6 bpm compared to 81.3 bpm in the non-yoga group, with a mean difference of −5.7 bpm (standard error = 3.0, 80% CI: [−9.6, −1.8], *p* value = 0.07). Other secondary results, including activity, sleep cycle, sleep efficiency, temperature, blood pressure, weight, and BMI, did not differ significantly between the two groups.

### 3.4 Safety measures

In the yoga group, 9.5% (2 of 21) reported adverse events, compared to 13.6% (3 of 22) in the non-yoga group. All adverse events were COVID-19 infections and were determined not to be attributable to the yoga intervention. No other serious adverse events or side effects were reported.

## 4 Discussion

This is the first study to investigate the efficacy of a yoga intervention for children exhibiting school refusal behavior using a cluster randomized controlled trial design to minimize the effects of institutional cross-contamination. This study evaluated the effectiveness of mindfulness yoga as an adjunct to traditional CBT for children exhibiting school-refusal behavior. The overall SCAS-C scores across the study participants demonstrated a significant decrease when analyzed using mixed-effects modeling. Notably, the anxiety reduction was particularly evident between the initial and final visits, thereby demonstrating the potential benefits of the intervention over time. On the other hand, the mindfulness yoga intervention did not significantly reduce anxiety levels among children with school refusal compared to treatment as usual. Although specific improvements were observed in the SCAS-C subscale for fears of physical injury and pulse rate in the yoga group, no significant differences were found in other subscales or secondary outcomes, such as psychosomatic tests or daily activity. The physical injury subscale of SCAS-C measures anxiety and fear levels stemming from specific phobias. Studies examining the relationship between specific phobia-induced fears and brain functional connectivity suggest that fear arousal may impair emotional regulation processes (Stefanescu et al., 2018). The reduction in fear of physical injury observed in this study may be associated with enhanced emotional regulation skills resulting from the sense of security gained through yoga practice. Furthermore, as indicated by previous research, the decrease in pulse rate can be attributed to the physiological calming effects induced by yoga practice (Kaleeswari et al., 2021). These findings imply that while yoga may offer specific advantages in certain areas, it does not outperform standard treatments in reducing overall anxiety levels and other psychological and behavioral outcomes in children with school refusal.

There were no significant improvements in daily activity levels in the yoga group. Our finding is consistent with broader research that, although acknowledging the general benefits of mindfulness and yoga on wellbeing and stress reduction, notes less consistent effects on anxiety-related behaviors such as school refusal. For example, Weaver & Darragh observed in their systematic review that yoga generally reduces anxiety among children and adolescents, however, its effectiveness can vary greatly depending on the population and setting, pointing to the need for more focused research (Weaver and Darragh, 2015). The lower attendance of the yoga group may be attributed to the limited short-term positive impact of yoga on attendance or the influence of other factors such as mental stress and home environment. Therefore, a larger sample size and longer study period, adjusted for other confounding factors such as home environment, would be needed to verify statistical significance of long-term effects of yoga.

Our study supports the potential advantages of a combined therapeutic approach that includes mindfulness yoga alongside more targeted treatments such as CBT. For instance, Randye et al. demonstrated that mindfulness training could improve self-management of attention in anxious children, indicating potential benefits when integrated with cognitive strategies targeting specific anxiety triggers (Semple et al., 2005). Additionally, a positive aspect of this study is the favorable safety profile of the mindfulness yoga intervention, which documents a low incidence of adverse events and positions it as a suitable option for pediatric populations where safety is a priority. This supports previous reports highlighting the value of yoga in improving mental health as well as providing important psychophysiological health benefits, especially in the treatment of pediatric psychiatric disorders where tolerability and patient safety are important (Khalsa, 2013).

The treatment status of children who refuse to attend school, particularly through the application of CBT, has been well documented and has shown significant efficacy. CBT has consistently shown improvements in school attendance and emotional wellbeing in children who have refused to attend school, and these benefits are maintained in follow-up studies (King et al., 1998). CBT tailored to the developmental needs of youth, such as the school project, has proven effective in increasing youth enrollment and reducing emotional symptoms (Sauter et al., 2009; Sauter et al., 2010). Similarly, successful outcomes have been reported with the use of CBT for children from marginalized and traumatized backgrounds (Blumkin, 2016). In cases where anxiety-based symptoms predominated, a combined approach of CBT and dialectical behavior therapy was beneficial; Lall found this combination to be effective in managing school refusal, reducing anxiety, and improving family dynamics (Lall, 2020). CBT has also been successfully applied to school-based interventions. Intensive exposure therapy, as demonstrated by Maeda et al., effectively treats 14-year-olds who refuse to attend school, demonstrating the potential of a school-based approach (Maeda et al., 2012). In addition, research supports the use of CBT in combination with pharmacological treatments. For example, the combination of CBT and fluoxetine was found to be superior to CBT alone in treating school refusal (Wu et al., 2013). Overall, although RCT evaluating the effectiveness of CBT are limited and have had only partial success, CBT remains the foundation of treatment for school refusal and often requires an individual approach, to addressing each child’s specific needs and underlying problems. The effectiveness of treatment for children struggling with school refusal can be increased by tailoring interventions to these needs, incorporating school-centered and family-centered strategies, and combining treatment modalities.

Although specific evidence is limited, yoga and mindfulness practices may offer various benefits for children who are reluctant to attend school. A study examining a 16-week integrated yoga and mindfulness program on the autonomic nervous system of elementary students found no significant changes in heart rate variability parameters (Ivaki et al., 2021). However, there was a tendency for increased parasympathetic activity in the intervention group (Ivaki et al., 2021). Britton et al. explored the effects of classroom-based mindfulness meditation on the mental health of 6th graders and found potential benefits, such as reduced suicidal thoughts (Britton et al., 2014). Another study indicated that implementing yoga and meditation in schools can reduce stress levels, improve attention spans, and enhance academic performance among children (Valentini and Raschi, 2023). McCabe et al. reported that elementary students found school-based mindfulness programs enjoyable and relaxing and were motivated to recommend them to others (McCabe et al., 2017). Additional research demonstrated that yoga positively impacts children’s stress management, academic success, and overall wellbeing (Tikhe and Ramarao, 2011). These studies suggest that yoga and mindfulness programs support children’s mental and physical health, stress management, and academic performance, while also being well-received by students. This indicates promising potential for incorporating yoga and mindfulness into school refusal behaviors.

It should be noted that this study is subject to several limitations that must be considered when interpreting the results. The impact of this study is limited by several factors, including its small sample size and the brief duration of the intervention. These limitations may have adversely affected our ability to detect significant differences between the intervention and control groups. Additionally, the analysis did not account for various social factors that could contribute to absenteeism, such as socio-economic background, parent–child relationships.

Additionally, the analysis did not account for various social factors that could contribute to absenteeism, such as socio-economic background, parent–child relationships, family support for yoga practice, institutional differences beyond free schools/non-free school facilities distinction, economic issues, and peer relationships. It should be noted that the exclusion criteria did not include developmental disorders, which represents another limitation.

Furthermore, challenges remain regarding the acceptability and adherence to the mindfulness yoga program. Despite implementing clear and careful introduction to yoga and its benefits, and maintaining motivation through regular interviews and telephone consultations, these efforts may not have sufficiently enhanced program acceptability, potentially limiting its effectiveness. The study was designed with minimal monitoring based on the premise that reducing psychological burden would maximize yoga’s benefits. However, this minimal monitoring may have decreased participant’s engagement to the yoga practice and dampened intervention effects, which represents theoretical and ethical limitation of the study. The fact that the study was conducted during the pandemic situation of COVID-19 had a significant impact on this study, which made their parents anxious about infection and refrained from entering the hospital influenced withdrawal. The irregularity of the days when school should be canceled due to the influence of the infection situation in society also had a significant impact on the children’s school attendance, mental symptoms, and rhythm of life. Future research could benefit from extended intervention period and larger participant cohorts to evaluate the impacts of mindfulness yoga more effectively, with consideration of in-person sessions and hybrid delivery models combining video and in-person sessions. Additionally, future studies should incorporate objective physiological measures such as heart rate variability and cortisol levels, addressing the current study’s limitations of lacking blinded evaluators and relying on potentially biased questionnaire-based primary outcomes.

Although the mindfulness yoga intervention did not markedly affect anxiety levels or school attendance in children with school refusal, limiting the generalizability of results, its safe profile encourages further investigation in pediatric contexts. Future studies should integrate mindfulness yoga with other therapeutic approaches and assess their collective impact over extended periods to thoroughly evaluate their potential benefits, which is essential for developing comprehensive, effective treatment strategies for complex pediatric conditions like school refusal.

## 5 Conclusion

This study evaluated the efficacy of a 4-week mindfulness yoga program in reducing anxiety among children with school refusal behavior. The findings suggest that while mindfulness yoga significantly reduces fears of physical injury and pulse rate in an exploratory sense, it does not affect substantially other psychological or behavioral outcomes such as school attendance and daily activity levels. Given its safety profile, mindfulness yoga can be considered a valuable complementary therapy to traditional treatments like CBT. However, further research is needed to explore its long-term effects, the most effective integration methods with other therapies, the mechanisms through which it exerts its effects, and to identify which subgroups of children may benefit the most from this intervention.

## Data Availability

The original contributions presented in the study are included in the article/Supplementary material, further inquiries can be directed to the corresponding author.
