# Supplementary figures and images for: An exploratory cluster-randomized controlled trial on mindfulness yoga’s effectiveness in school-refusing children: reductions in SCAS-C physical injury fears and pulse rate
Source: Front Hum Neurosci. 2024 Dec 11;18:1468729. doi: 10.3389/fnhum.2024.1468729 (PMC11683735; doi:10.3389/fnhum.2024.1468729)

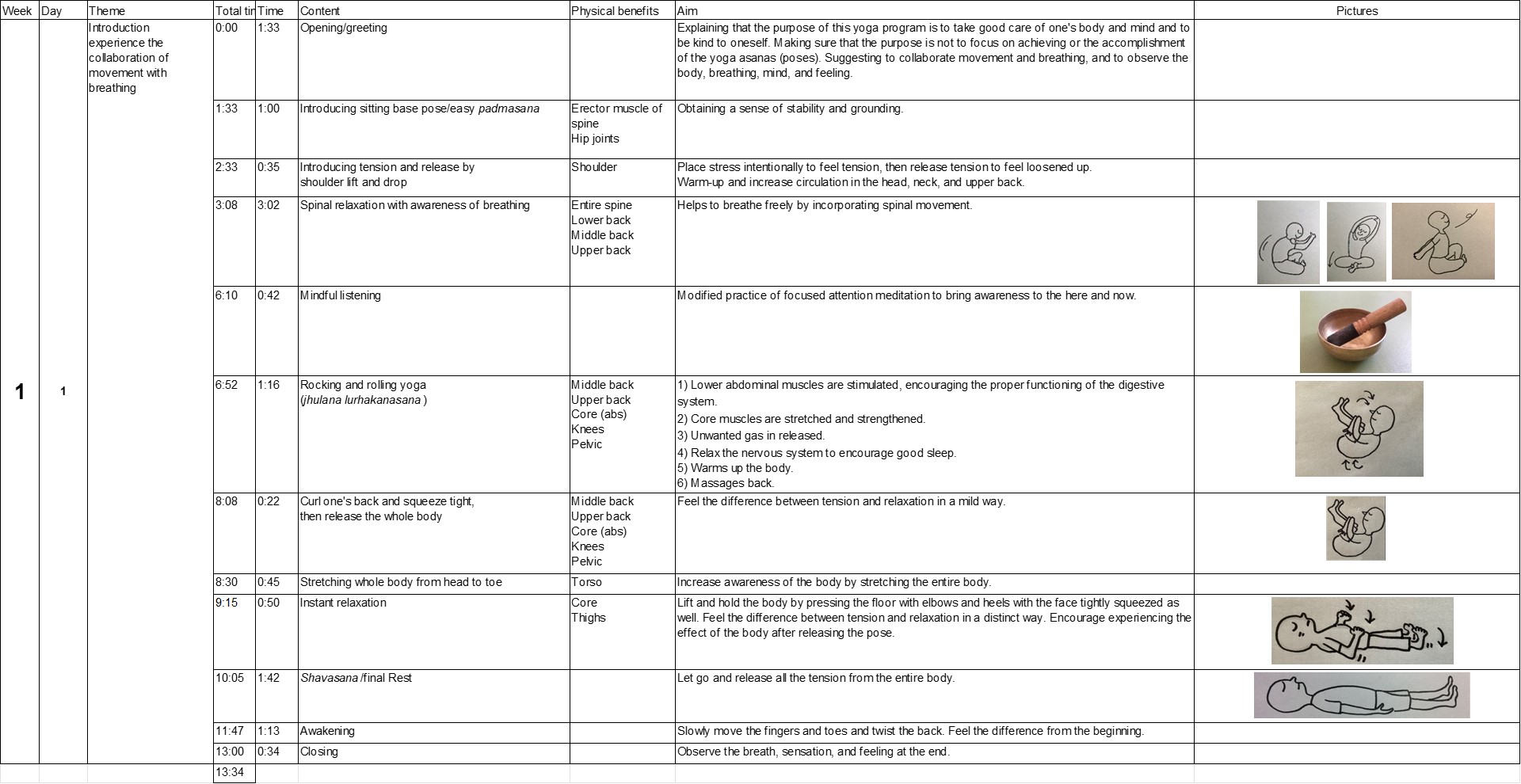

Supplement: Supplementary file 1 [file Image_1.JPEG]

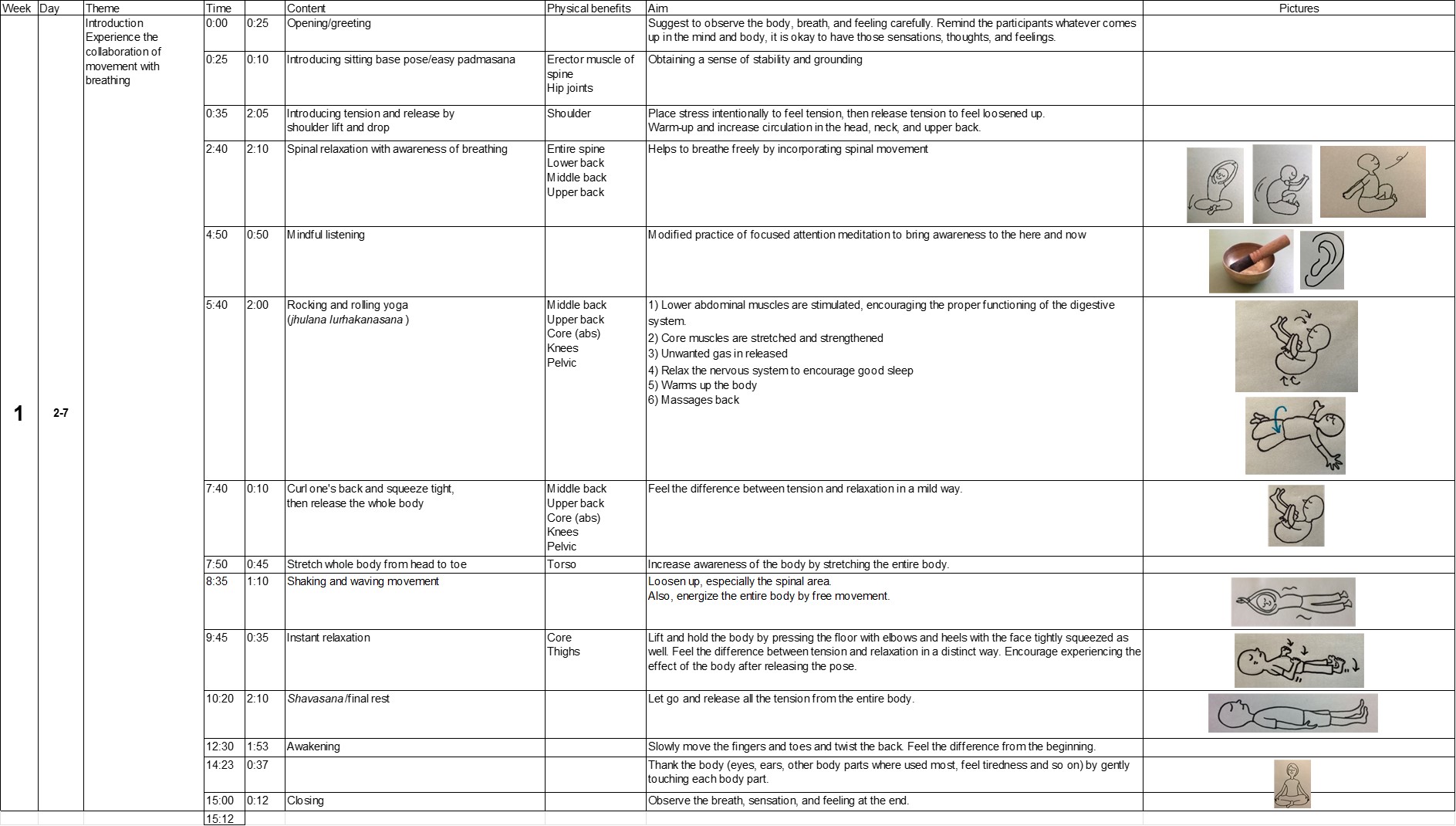

Supplement: Supplementary file 2 [file Image_2.JPEG]

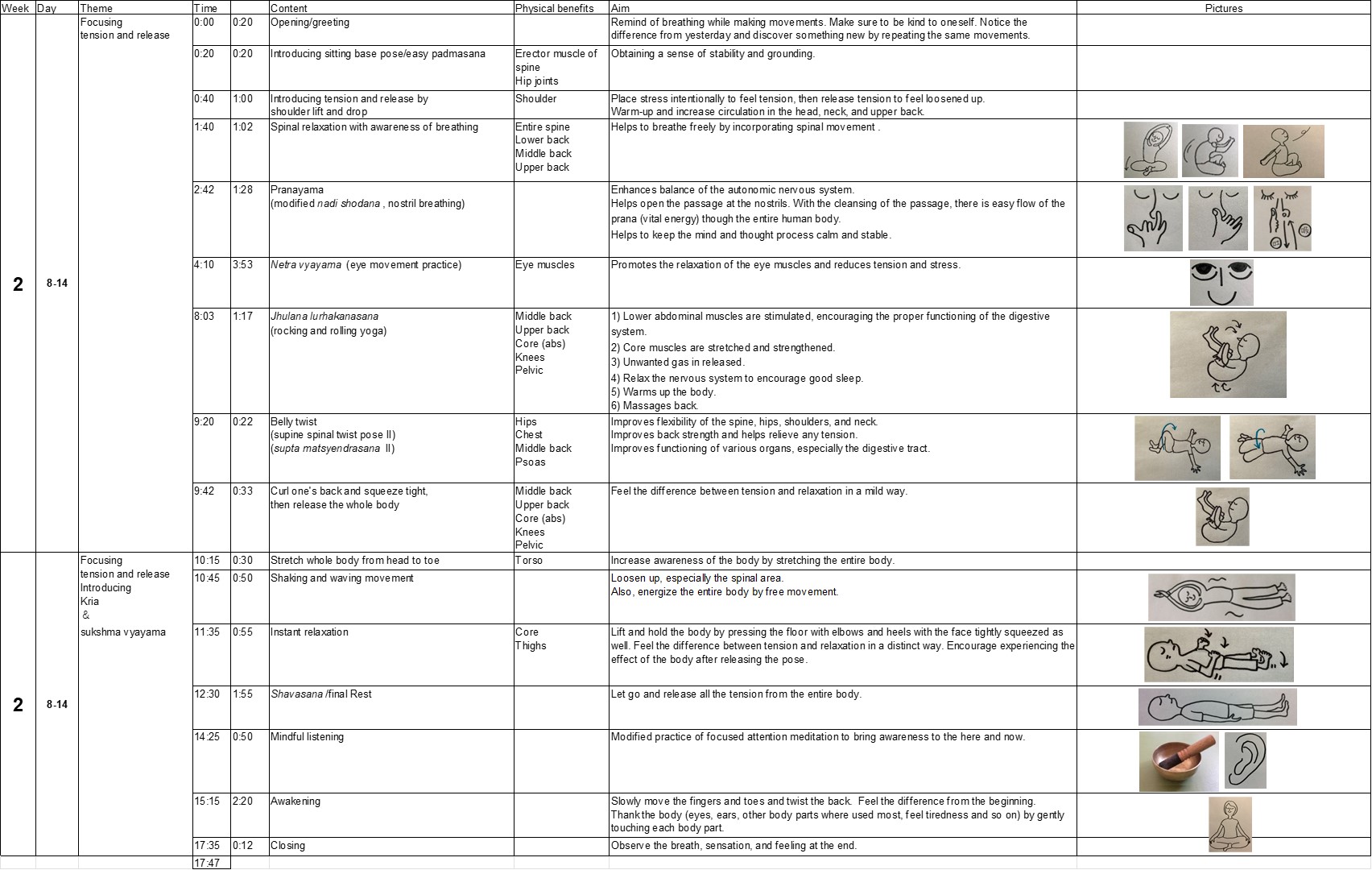

Supplement: Supplementary file 3 [file Image_3.JPEG]

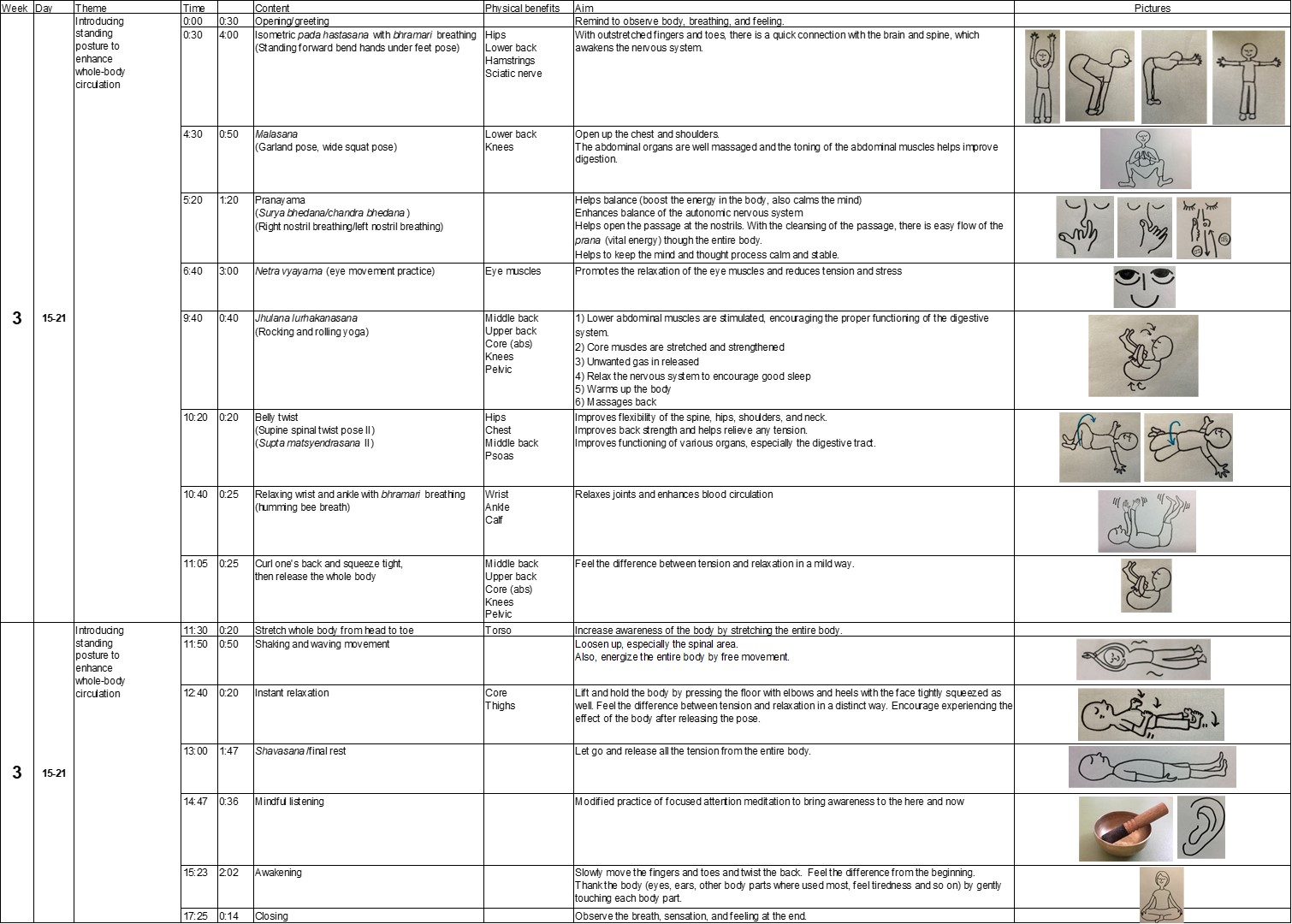

Supplement: Supplementary file 4 [file Image_4.JPEG]

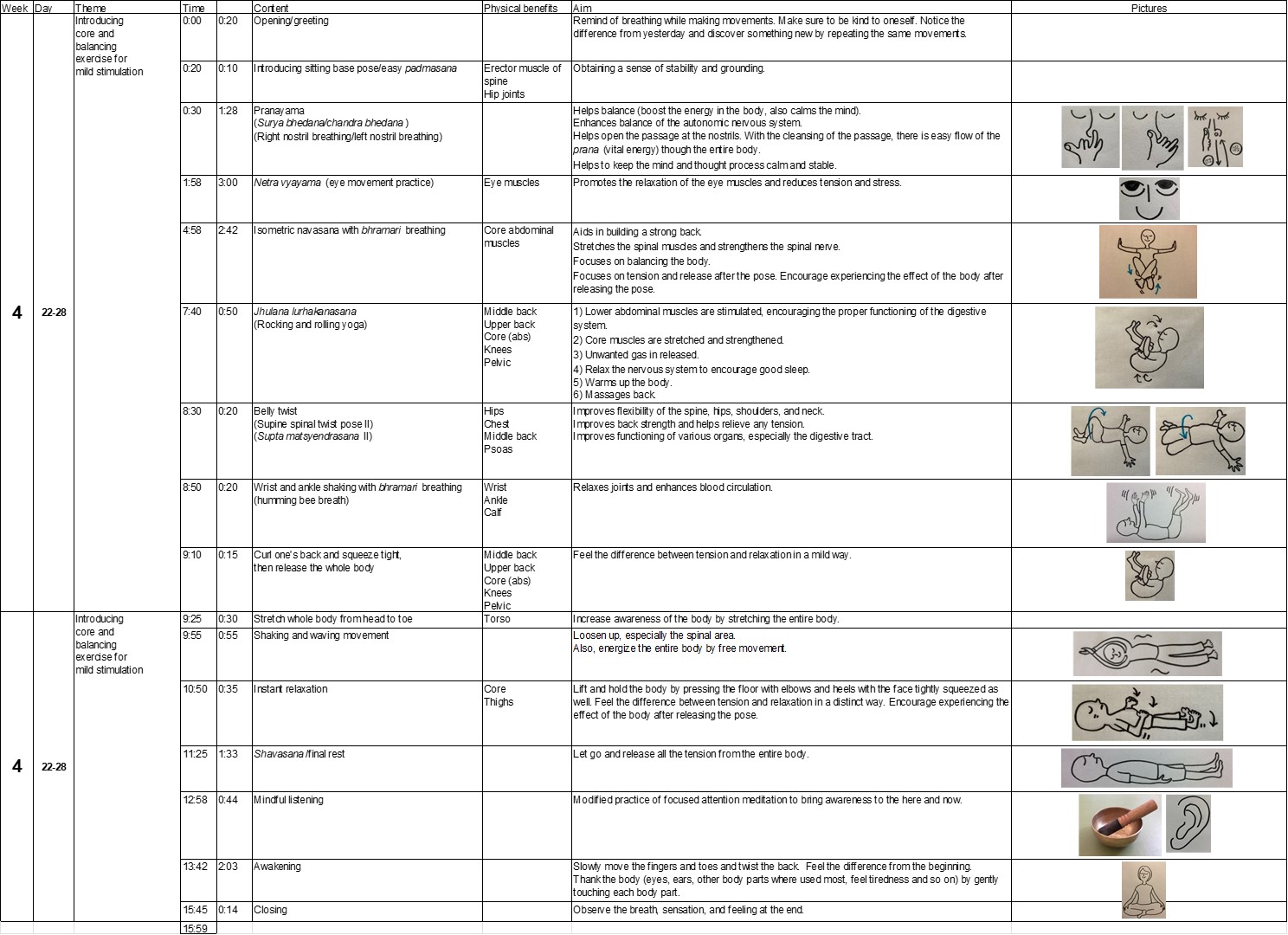

Supplement: Supplementary file 5 [file Image_5.JPEG]

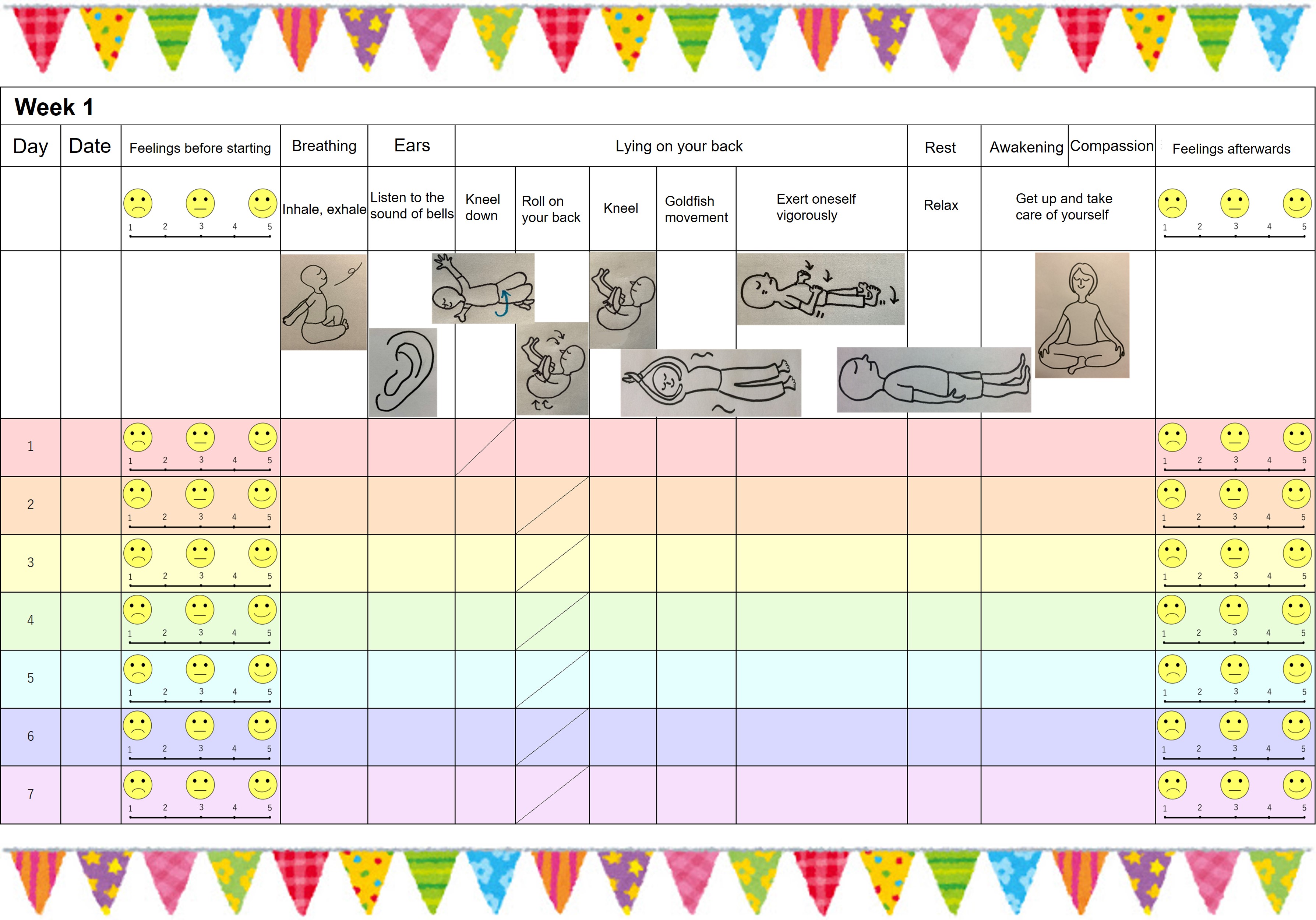

Supplement: Supplementary file 6 [file Image_6.JPEG]

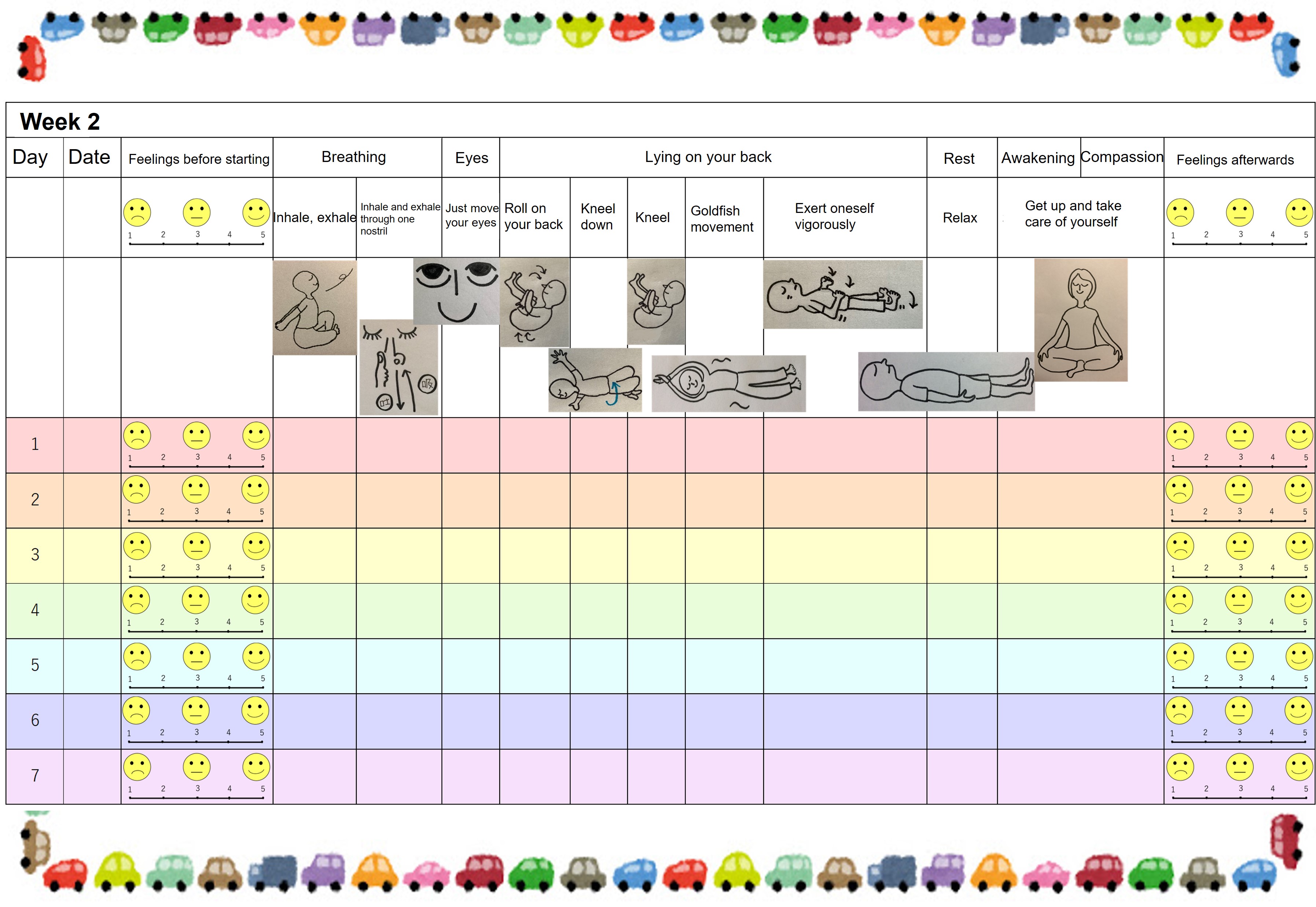

Supplement: Supplementary file 7 [file Image_7.JPEG]

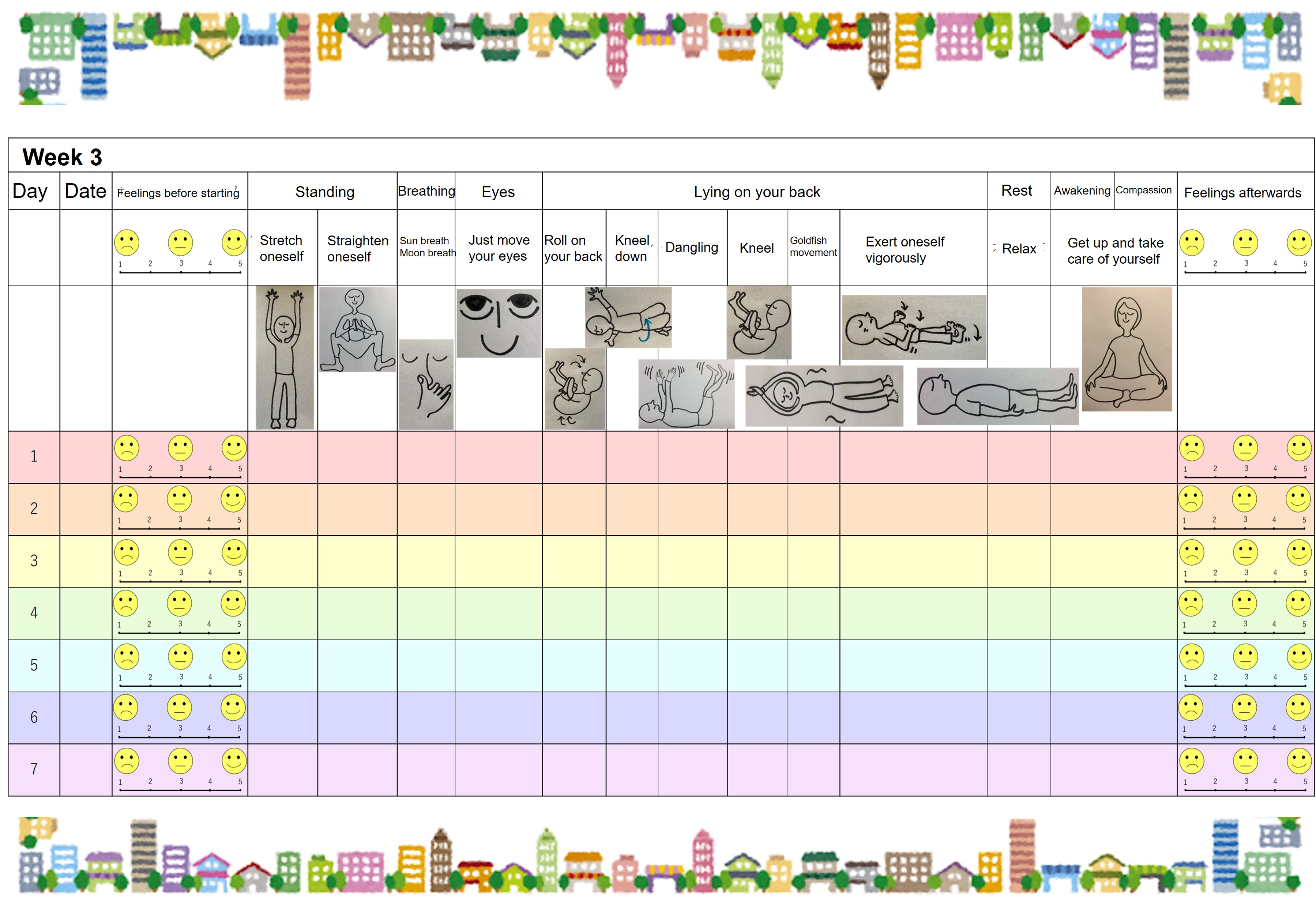

Supplement: Supplementary file 8 [file Image_8.JPEG]

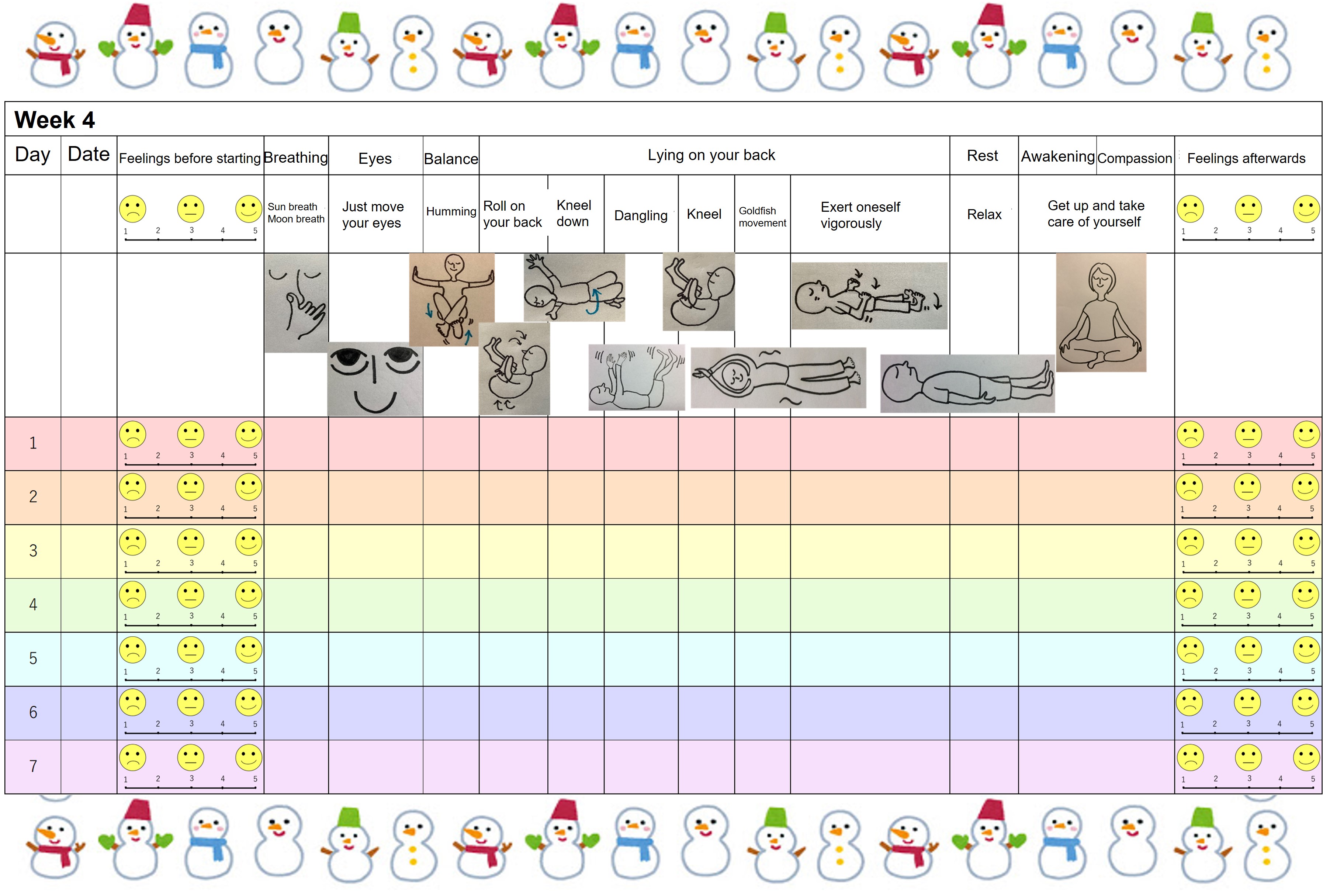

Supplement: Supplementary file 9 [file Image_9.JPEG]
